# Dynamics of Serum Retinol and Alpha-Tocopherol Levels According to Non-Alcoholic Fatty Liver Disease Status

**DOI:** 10.3390/nu13051720

**Published:** 2021-05-19

**Authors:** Dongsub Jeon, Minkook Son, Juhyun Shim

**Affiliations:** 1 Liver Center, Asan Medical Center, Department of Gastroenterology, University of Ulsan College of Medicine, Seoul 05505, Korea; thinkdoc87@hanmail.net; 2 Department of Biomedical Science and Engineering, Gwangju Institute of Science and Technology, Gwangju 61005, Korea; minkook87@naver.com

**Keywords:** non-alcoholic fatty liver disease, vitamin A, vitamin E, advanced liver fibrosis

## Abstract

The available data on the association between micronutrients in the blood and non-alcoholic fatty liver disease (NAFLD) are limited. To investigate the clinical implications of this relationship, we sought to identify the difference in the serum levels of vitamins A and E according to NAFLD status using data from the seventh Korea National Health and Nutrition Examination Survey. In this cross-sectional study of the Korean population, NAFLD and its severity were defined using prediction models. Differences in the prevalence and severity of NAFLD were analyzed according to serum retinol (vitamin A) and alpha (α)-tocopherol (vitamin E) levels. Serum levels of retinol and α-tocopherol were positively correlated with the prevalence of NAFLD. In most prediction models of the NAFLD subjects, serum retinol deficiency was significantly correlated with advanced fibrosis, while serum α-tocopherol levels did not differ between individuals with or without advanced fibrosis. Similar trends were also noted with cholesterol-adjusted levels of α-tocopherol. In summary, while circulating concentrations of retinol and α-tocopherol were positively associated with the presence of NAFLD, advanced liver fibrosis was only correlated with serum retinol levels. Our findings could provide insight into NAFLD patient care at a micronutrient level.

## 1. Introduction

Non-alcoholic fatty liver disease (NAFLD) is the most common liver-related disorder globally. It is currently present in approximately 25% of the world’s population, and this figure continues to rise due to the increased prevalence of obesity and the aging population [1]. NAFLD varies from simple hepatic steatosis without inflammation or fibrosis to non-alcoholic steatohepatitis (NASH), which can lead to cirrhosis and hepatocellular carcinoma [2]. Therefore, effectively preventing and treating NAFLD has become increasingly necessary due to the clinical and economic burden of the disease. To do this, diagnosing NAFLD early is crucial. To meet the practical need for noninvasive but accurate methods to diagnose NAFLD and predict the risk of NASH, numerous studies have investigated prediction models using single or combined biochemical and/or anthropometric parameters [3,4,5,6,7,8]. Various hepatic steatosis formulae for the diagnosis of NAFLD have proven comparable to standard ultrasonography in multiple cohorts [5,9,10,11], and a recent meta-analysis also reported acceptable performance of the simple biological scoring systems, including the BMI-AST/ALT Ratio-Diabetes (BARD) score and the Fibrosis-4 (FIB-4) index, when compared to transient elastography in the detection of advanced fibrosis [12,13,14,15].

Among the known factors associated with NAFLD, the pathogenic role of macronutrients is well-established in NAFLD. However, the contribution of micronutrients to NAFLD pathogenesis has garnered less attention than to obesity [16]. Vitamins are essential micronutrients that regulate cellular growth and metabolism and are categorized as either fat-soluble or water-soluble. NAFLD may disturb the metabolism of fat-soluble vitamins (A, D, E, and K) [17]. Retinoic acid, an active metabolite of vitamin A, is associated with the pathogenesis of NAFLD through its effect on hepatic lipid metabolism and insulin resistance [18]. Vitamin E, another fat-soluble vitamin, has antioxidant, anti-inflammatory, and antifibrotic effects [19].

Interestingly, findings on serum levels of vitamin A and E in NAFLD are controversial. According to Mazidi et al.’s cross-sectional study using the Nutrition and Health Examination Surveys data, serum vitamin A and E levels are negatively associated with NAFLD severity [20]. On the other hand, in a cross-sectional study conducted by Pettinelli et al., serum retinol levels were higher in NAFLD patients than in healthy liver donors without NAFLD [21]. Additionally, serum alpha (α)-tocopherol levels were positively associated with central adiposity after adjusting for body fat in another cross-sectional study [22].

Recently, a study concluded that high serum retinol and α-tocopherol levels were associated with an increased risk of metabolic syndrome in South Korean patients [23]. Given that NAFLD is a hepatic manifestation of metabolic syndrome, we hypothesized positive correlations between the vitamin concentrations and NAFLD. Since vitamin A is metabolized and maintains its homeostasis in the liver, NAFLD could have a more direct influence on vitamin A homeostasis than metabolic syndrome has [18]. Additionally, since vitamin E is used as a treatment for NAFLD accompanied by NASH, monitoring and maintaining vitamin E status in NAFLD patients is essential [24].

Therefore, we decided to conduct a large population-based study using public data on 4448 subjects from South Korea to determine the association of serum retinol and α-tocopherol levels according to disease status in subjects with NAFLD diagnosed by noninvasive laboratory methods and to provide an informative reference for the nutritional and clinical management of patients.

## 2. Materials and Methods

### 2.1. Data Information and Study Population

The KNHANES (Korea National Health and Nutrition Examination Survey) is a cross-sectional survey and a nationally representative database of Korea managed by the Korea Centers for Disease Control and Prevention (KCDCP) [25]. This study used data from the seventh KNHANES VII (2016–2018) [26]. The KNHANES database includes the results of physical examinations, laboratory tests, health-related interviews, and nutritional surveys. Informed consent was acquired from subjects for their voluntary participation, and the KNHANES has been approved by the Institutional Review Board of the KCDCP. This study’s protocol was reviewed and approved by the Institutional Review Board of Asan Medical Center (IRB number: 2020–1893), which waived the need for informed consent because the data were anonymized and analyzed retrospectively. This study was performed in accordance with the Declaration of Helsinki.

**Figure 1:**
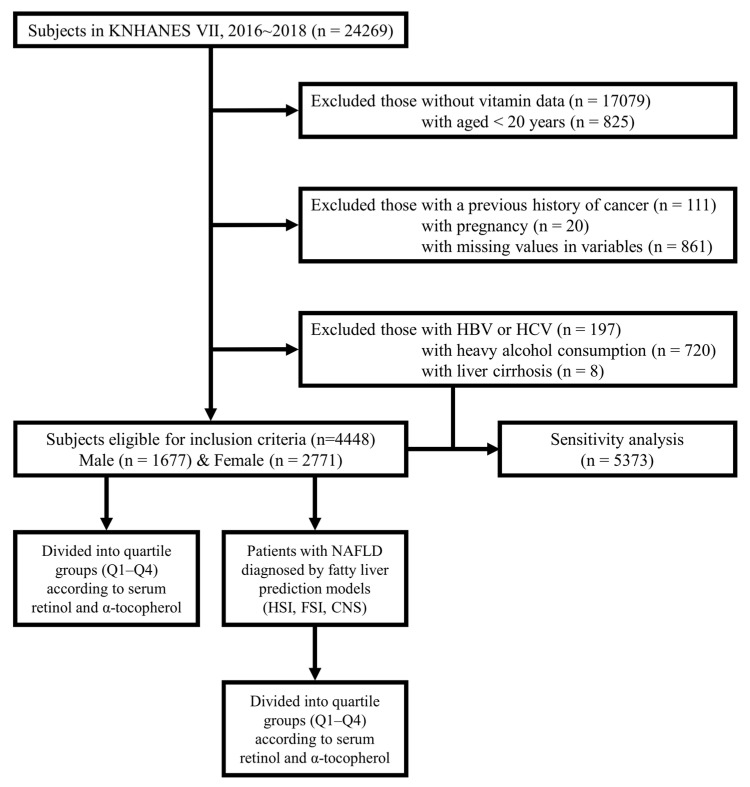
Flow diagram of study population. From the total 24,269 subjects retrieved from the KNHANES VII database, we excluded the following subjects: (1) 17,079 subjects without data on serum retinol and alpha (α)-tocopherol levels; (2) 825 aged < 20 years; (3) 111 with a previous history of any cancer; (4) 20 who were pregnant; and (5) 861 with missing variables. An additional 926 subjects, (1) 197 positive for HBV or HCV; (2) 720 heavy drinkers; and (3) 8 with liver cirrhosis were also excluded from the initial analysis but included in the later sensitivity analysis. Finally, 4448 subjects (1677 males and 2771 females) were included. All included individuals were divided into quartile groups according to serum retinol and α-tocopherol levels. KNHANES VII, 7th Korea National Health And Nutrition Examination Survey; HBV, hepatitis B virus; HCV, hepatitis C virus; Q, quartile; NAFLD, non-alcoholic fatty liver disease; HSI, hepatic steatosis index; FSI, Framingham steatosis index; CNS, comprehensive NAFLD score.

From the total 24,269 subjects retrieved from the KNHANES Ⅶ database, we excluded 17,079 subjects without serum retinol and α-tocopherol levels. We excluded those aged <20 years (*n* = 825), those with a previous history of any cancer (*n* = 111), those who were pregnant (*n* = 20), and those with missing variables (*n* = 861). Additionally, subjects who met the following criteria were excluded from the initial analysis: (a) those with positive markers for hepatitis B virus (HBV) or hepatitis C virus (HCV) (*n* = 197), (b) severe alcoholics with daily drinking (*n* = 720), and (c) those with liver cirrhosis (*n* = 8). This excluded population (*n* = 925) was later included in the study population for a sensitivity analysis. Finally, this study comprised 4448 subjects (1677 males and 2771 females). The study population was divided into quartile groups according to serum retinol and α-tocopherol levels. Subsequently, using fatty liver prediction models, the patients with NAFLD were further divided into quartile groups according to serum retinol and α-tocopherol levels. A flow diagram for this study is presented in Figure 1.

### 2.2. Clinical Variable Measurements and Laboratory Analyses

Anthropometric measurements were performed by trained staff. The body mass index (BMI) was calculated using the formula weight (kg) divided by height squared (m^2^). Subjects were categorized as obese if their BMI was ≥25 kg/m^2^ and as non-obese if the BMI was <25 kg/m^2^ [27].

All subjects fasted for at least 8 h prior to taking blood samples. The blood samples were refrigerated immediately for transportation to the Central Testing Institute (NeoDin Medical Institute, Seoul, Korea). Fasting glucose, total cholesterol, triglycerides, high-density lipoprotein cholesterol (HDL-C), and creatinine were measured by the hexokinase UV, enzymatic, homogeneous enzymatic colorimetric, Jaffe rate-blanked, and compensated methods, respectively, using the Hitachi Automatic Analyzer 7600-210 (Hitachi, Tokyo, Japan). For the quantification of serum retinol and α-tocopherol levels, high-performance liquid chromatography-fluorescence with Agilent 1200 (Agilent, Santa Clara, CA, USA) was applied using Chromsystems (Chromsystems Instruments & Chemicals, Munich, Germany). The normal reference range for serum retinol levels in adults is 0.30–0.70 mg/L, and for serum α-tocopherol levels in adults, it is 5.00–20.00 mg/L. Further information on the blood tests is described on the KCDCP website [25]. The estimated glomerular filtration rate (eGFR) was determined from the Modification of Diet in Renal Disease equation [28].

Hypertensive status was divided into three categories: (a) hypertension was defined as systolic blood pressure (SBP) ≥ 140 mmHg or diastolic blood pressure (DBP) ≥ 90 mmHg or taking anti-hypertensive medication; (b) pre-hypertension was defined as 120 mmHg ≤ SBP < 140 mmHg or 80 mmHg ≤ DBP < 90 mmHg; and (c) normal was defined as SBP < 120 mmHg and DBP < 80 mmHg [29]. Diabetic status was divided into three groups: (a) diabetes was defined as fasting blood glucose (FBG) ≥ 126 mg/dL or taking anti-diabetic medication; (b) pre-diabetes was defined as 100 mg/dL ≤ FBG < 126 mg/dL; and (c) normal was defined as FBG < 100 mg/dL [30]. Dyslipidemia status was categorized into two groups using the dyslipidemia criteria for Koreans, with cutoff values as follows: total cholesterol ≥ 240 mg/dL, triglycerides ≥ 200 mg/dL, or HDL-C ≤ 40 mg/dL [31].

Demographic and medical information was obtained from self-report questionnaires and face-to-face interviews with trained staff. Income level was classified into four groups. Smoking status was categorized into current smokers, ex-smokers, and non-smokers. Alcohol consumption status was categorized into two groups: (a) non-consumers, with no alcohol intake in the past year or less than once a month, and (b) alcohol consumers, with alcohol intake more than once a month. Physical exercise status was categorized into two groups: (a) the regular exercise group, with moderately intense exercise at least 150 min within a week, intense exercise at least 75 min within a week, or mixed exercise equivalent to the above level (1 min of intense exercise equivalent to 2 min of moderately intense exercise), and (b) the non-regular exercise group, with physical activity less than the level described above [32].

Dietary information was investigated with the 24-hour recall method. According to the Dietary Reference Intakes for Koreans criteria, vitamin A was measured using retinol equivalents (RE) and derived from the following equation: RE (μg) = retinol (μg) + β-carotenes/6 (μg) [33]. However, information on vitamin E consumption was not included in the KNHANES VII database. Use of vitamin supplements was categorized into non-supplement users (no vitamin supplements) and supplement users (vitamin supplements for more than two weeks within a 1-year period).

### 2.3. Definitions of Hepatic Steatosis and Advanced Fibrosis

NAFLD was defined using the following fatty liver prediction models: (a) the hepatic steatosis index (HSI), (b) the Framingham steatosis index (FSI), and (c) the comprehensive NAFLD score (CNS) [4,5,34]. The BARD and FIB-4 scores were applied to define advanced fibrosis and were calculated in subjects classified as having NAFLD using the aforementioned prediction models [14,15]. All prediction models are described in Table S1.

### 2.4. Statistical Analysis

The subjects’ characteristics were described according to vitamin (serum retinol and α-tocopherol) and NAFLD (HSI, FSI, and CNS) status. Continuous variables are reported as mean with standard deviation, and categorical variables are reported as number with percentage. Characteristics of the quartile groups for serum retinol and α-tocopherol were compared using the one-way analysis of variance and the chi-square test. The association between NAFLD status and vitamin (serum retinol and α-tocopherol) levels was estimated using logistic regression models. The model for serum retinol was adjusted for age, sex, BMI, GFR, hypertension, diabetes, dyslipidemia status, income level, smoking status, alcohol consumption, exercise status, use of vitamin supplements, and daily dietary intake of vitamin A. For the model for α-tocopherol, all the aforementioned variables except the daily dietary intake of vitamin A, were included for adjustment. Subgroup analyses were performed according to the presence of obesity and diabetes. The association between advanced fibrosis (BARD and FIB-4) status and vitamin (serum retinol and α-tocopherol) levels was estimated using the same multivariable-adjusted logistic regression models. Moreover, we performed an additional analysis with α-tocopherol adjusted for cholesterol (α-tocopherol/cholesterol ratio) because α-tocopherol can be affected by lipid metabolism [35]. Data collection and statistical analyses were performed with SPSS software version 20 (IBM, SPSS Inc., Chicago, IL, USA). The *p*-value < 0.05 was considered statistically significant.

## 3. Results

### 3.1. Baseline Characteristics of Study Subjects

**Figure 2:**
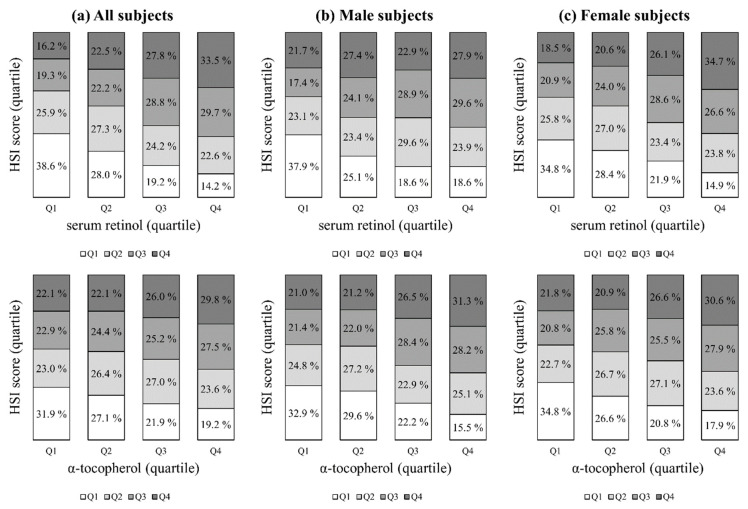
Proportion of subjects with serum retinol and α-tocopherol levels according to quartile with predicted NAFLD prevalence (HSI) by quartile. The study population was divided into quartile groups according to NAFLD (HSI) and vitamin (serum retinol and α-tocopherol) levels, respectively. Serum retinol and α-tocopherol had strong positive associations with the NAFLD (HSI) prediction model (*p*s < 0.0001 for the trends). NAFLD, non-alcoholic fatty liver disease; HSI, hepatic steatosis index; Q, quartile.

The baseline characteristics of the study subjects are summarized according to the quartile groups for serum retinol and α-tocopherol in Table 1 and Table 2, respectively. As serum retinol and α-tocopherol increased, the mean age, SBP, BMI, FBG, total cholesterol, and triglycerides increased, but the mean HDL-C and GFR decreased. The subjects in the higher quartiles for serum retinol and α-tocopherol were more likely to be male; had a greater prevalence of hypertension, diabetes, and dyslipidemia; were more likely to be current smokers and alcohol consumers; and were more likely to use vitamin supplements. There were no significant differences in the proportion who exercised regularly or in the daily dietary intake of vitamin A. The mean values of serum retinol according to quartile from Q1 to Q4 were 0.32 mg/L, 0.43 mg/L, 0.53 mg/L, and 0.74 mg/L, respectively, while the mean values of α-tocopherol were 8.7 mg/L, 11.4 mg/L, 13.9 mg/L, and 20.3 mg/L, respectively. As the quartile of serum retinol and α-tocopherol increased, the prevalence of NAFLD assessed according to the HSI, FSI, and CNS also increased. The proportions of patients with serum retinol and α-tocopherol levels according to quartile using different NAFLD scores by quartile are presented in Figure 2 and in Figures S1 and S2. In addition, the proportion of subjects with serum retinol levels according to the reference value of different NAFLD scores are presented in Figure S3. Serum retinol and α-tocopherol had strong positive associations with all NAFLD prediction models (*p*s < 0.0001 for the trends).

### 3.2. Association between Vitamins and NAFLD Assessed Using Different Prediction Models (HSI, FSI, CNS)

The multivariable adjustment was performed for variables that could affect the prevalence of NAFLD. The adjusted odds ratios (AORs) and 95% confidence intervals (CIs) for the association between the vitamins and NAFLD which was assessed using the different prediction models are shown in Table 3 and Table 4. Since there was a disparity for sex, we examined the association between the vitamins and NAFLD after stratification by sex. In all subjects, serum retinol and α-tocopherol were significantly associated with NAFLD regardless of the prediction model. Specifically, the AORs and 95% CIs for NAFLD comparing Q4 with Q1 were 1.67 (1.22–2.45), 2.34 (1.65–3.32), and 3.28 (2.33–4.61) for serum retinol and 1.44 (1.01–2.05), 3.96 (2.83–5.56), and 3.39 (2.44–4.71) for α-tocopherol in the HSI, FSI, and CNS models, respectively. In the analysis stratified by sex, there were significant associations between the vitamins and NAFLD for all prediction models, except for HSI in male subjects.

We also performed an additional sensitivity analysis on the subjects including those excluded from the primary analysis because they had HBV, HCV, liver cirrhosis, and severe alcoholism. The results, which were consistent with the primary results regardless of gender, are summarized in Tables S2 and S3. Moreover, the results of the additional analysis for the association between α-tocopherol with the cholesterol adjustment and NAFLD are presented in Table S4. As shown in the results, α-tocopherol with the cholesterol adjustment was significantly associated with NAFLD except for HSI in all subjects and in male subjects.

### 3.3. Subgroup Analysis According to Obesity and Diabetes

**Figure 3:**
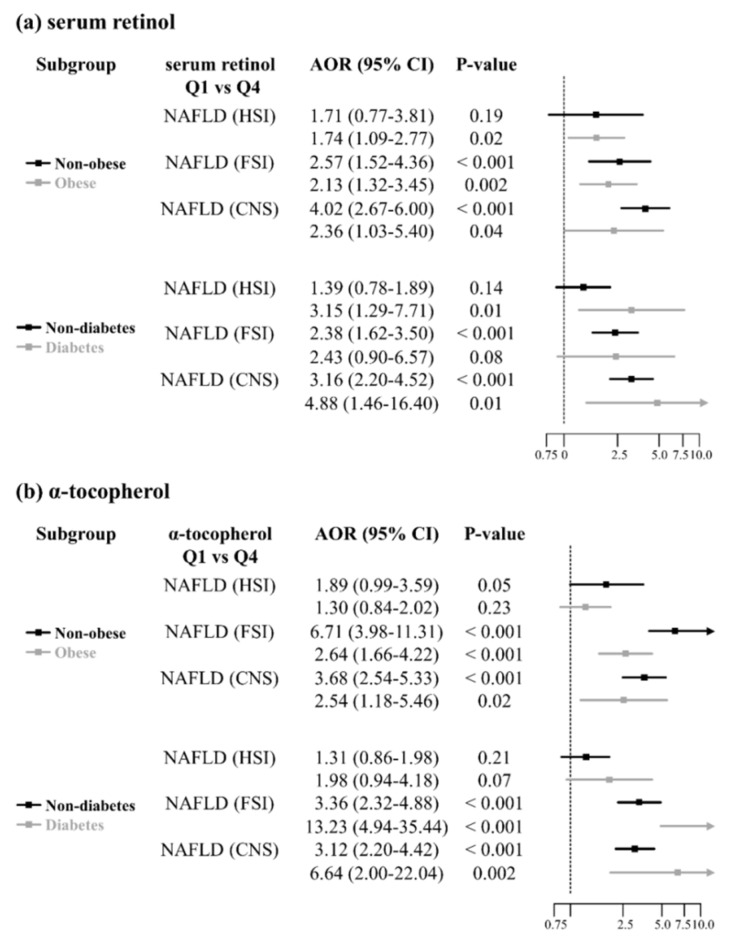
Subgroup analysis according to obesity and diabetes. The association between NAFLD status and vitamin (serum retinol and α-tocopherol) levels was estimated using logistic regression models. The logistic model was adjusted for age, sex, BMI, GFR, hypertension, diabetes, dyslipidemia status, income level, smoking status, alcohol consumption, exercise status, use of vitamin supplements, and daily dietary intake of vitamin A. The association remained significant for NAFLD according to FSI and CNS, except in diabetic patients according to FSI. Q, quartile; AOR, adjusted odds ratio; CI, confidence interval; NAFLD, non-alcoholic fatty liver disease; HSI, hepatic steatosis index; FSI, Framingham steatosis index; CNS, comprehensive NAFLD score.

Since obesity and diabetes are well-established risk factors for NAFLD, we analyzed the association between the vitamins and NAFLD after dividing the subgroups according to obesity and diabetes. The AORs and 95% CIs for NAFLD comparing Q4 with Q1 for serum retinol and α-tocopherol are summarized in Figure 3. According to the HSI prediction model, the association between the vitamins and NAFLD was either weak or non-significant, while the association remained significant for NAFLD according to FSI and CNS, except in diabetic patients according to FSI. The forest plot showed that NAFLD prevalence was positively associated with serum retinol and α-tocopherol levels, especially in non-obese or diabetic subjects.

### 3.4. Association between Vitamins and Advanced Fibrosis Assessed Using Different Prediction Models (BARD, FIB-4)

When we examined the association between the vitamins and advanced fibrosis, we calculated the BARD and FIB-4 scores within the NAFLD population assessed by the three NAFLD prediction models. The proportions of patients with serum retinol levels according to the reference values for different advanced fibrosis scores are presented in Figure S4. The proportion of subjects with advanced fibrosis tended to decrease with increasing serum retinol levels in the analysis based on HSI and CNS, although the *p*-value for the trend was insignificant for FSI.

After serum retinol and α-tocopherol levels were classified into quartile groups, we performed a multivariable-adjusted logistic regression analysis, the results of which are presented in Table 5. Most AORs and 95% CIs for advanced fibrosis comparing Q1 for serum retinol to Q2, Q3, and Q4 were significant, although not within the NAFLD population assessed using the FSI model. Additionally, there was no significant association between α-tocopherol and advanced fibrosis, except for the finding from the BARD model in FSI-based NAFLD subjects. Additional analyses with cholesterol-adjusted levels of α-tocopherol did not show any significance for any of the associations (Table S5).

## 4. Discussion

This study investigated the association between serum vitamin A (retinol) and E (α-tocopherol) levels and the prevalence of NAFLD in Korean adults. The prevalence of NAFLD was positively associated with serum retinol and α-tocopherol levels independently from obesity and diabetes status. However, when patients with advanced fibrosis in the NAFLD group were evaluated, serum retinol levels and the presence of fibrosis tended to be inversely associated. Moreover, patients with serum retinol deficiency showed advanced fibrosis at a statistically higher frequency. Conversely, serum α-tocopherol levels were not associated with the progression of advanced fibrosis.

There have been mixed reports on serum levels of vitamin A and E in NAFLD [20,21,22]. This could be due to study designs that included patients with all types of NAFLD, ranging from simple hepatic steatosis to NASH. Additionally, the prediction models used to estimate NAFLD varied in previous studies. Mazidi et al., demonstrating different results from ours, used only the fatty liver index (FLI) to define NAFLD [20]. In a validation study on the Korean population, FLI showed the lowest diagnostic performance compared to four other prediction models with an area under the receiver operating characteristic curve of 0.68 [12]. Therefore, the results from this study showing a negative association of serum vitamin A and E levels with NAFLD should be interpreted with caution. In our study, we used three different prediction models to measure NAFLD to increase the robustness of the study. On the other hand, a German study of 57 biopsy-proven NASH patients reported that, compared to healthy controls, the disease was significantly associated with lower serum levels of α-tocopherol but not with lower serum levels of retinol [36]. The discrepancy between the prior study and ours may be due to the substantial differences in diagnostic criteria and the associated disease status.

Chen proposed a theory that a disturbance in vitamin A homeostasis in the liver may contribute to the development of NAFLD [37]. Specifically, he argued that in certain circumstances of the diseased state (i.e., changes in dietary or hormonal signals), hepatic stellate cells (HSCs) may lose their ability to store vitamin A in the liver [38]. Consequently, excessive vitamin A metabolism would occur, which could increase lipogenesis and contribute to fat accumulation in hepatocytes [39]. Additionally, the decrease in vitamin A storage in HSCs would induce more dietary vitamin A to be catabolized in the liver, resulting in the excessive release of retinol binding protein (RBP)/retinol complex [37]. This would act as a vicious cycle leading to a change in gene expression for glucose and lipid metabolism, eventually inducing NAFLD and NASH [40]. Based on our study results, it is speculated that, in the developmental stage and early phase of NAFLD, disturbed vitamin A homeostasis, which could affect lipid accumulation in the liver, results in elevated serum retinol levels due to an excessive release of RBP/retinol complex. As NAFLD progresses into advanced fibrosis, impaired vitamin A homeostasis causes severe vitamin A deficiency in the liver, resulting in a decrease in the serum retinol level [37]. This is consistent with a previous study investigating the association between vitamin A levels in the liver and NAFLD severity based on biopsy results: this study found that the more the hepatic vitamin A content decreased, the more NAFLD progressed [41]. However, this circumstance was not necessarily related to the plasma vitamin A level, given that serum retinol levels reflect hepatic vitamin A stores only when they are extremely deficient or excessive [42]. In sum, serum retinol deficiency in patients with NAFLD might be an indicator for fibrosis, although more data is needed to validate this association.

Only 1.1% of the subjects included in our study were deficient in vitamin E. As the serum α-tocopherol level increased, the risk of developing NAFLD increased. This is consistent with the in vivo study by Bartolini et al. that showed that impaired cytochrome P-450-dependent vitamin E metabolism in the NAFLD-induced rat model resulted in the accumulation of α-tocopherol in the liver [43]. Additionally, Nagita et al. found a correlation between hepatic and serum α-tocopherol levels [44]. Therefore, it is conceivable that high serum α-tocopherol levels were associated with NAFLD. In the subgroup analysis, serum α-tocopherol levels showed a stronger association with NAFLD in non-obese or diabetic subjects, whereas there was no significant difference in serum α-tocopherol levels between patients with simple steatosis without fibrosis and patients with advanced fibrosis, except for the finding from the BARD model in FSI-based NAFLD patients. These results have clinical implications because α-tocopherol is used as a treatment for patients with NAFLD who have NASH, and some safety issues have been found [45].

NAFLD is a progressive disease that varies from simple hepatic steatosis to NASH. Our study evaluated the dynamic changes in serum vitamin A and E levels in patients with NAFLD according to the severity of the disease. According to our knowledge, it is the first study to assess differences in serum vitamin A and E levels in healthy people without NAFLD, patients with NAFLD but without advanced fibrosis, and patients with NAFLD with advanced fibrosis using nationally representative data. As to vitamin A, we found a correlation between serum retinol deficiency and advanced liver fibrosis. This finding is noteworthy in that it could provide a reference for determining when to conduct a biopsy to rule out NASH, although more studies are needed on this issue. Additionally, since vitamin E is used as a treatment for patients with NAFLD who have NASH, these results could provide clinicians references for NAFLD patient care at a micronutrient level.

Despite the strengths of our study, there are also several limitations. First, even though serum retinol levels decreased in patients with NAFLD who had advanced fibrosis, we could not confirm differences in serum retinol levels among the NASH subclasses according to histopathologic severity since there were no biopsy results for NAFLD. Second, information on supplementary vitamin E intake was not included in the data, unlike supplementary vitamin A intake. This could have introduced bias into our results. Third, we used noninvasive prediction models to assess NAFLD or advanced fibrosis rather than biopsy or imaging test results because those were unavailable due to the nature of the data source. Such scoring diagnoses may be less precise than biopsy-confirmed results or imaging tests. Nonetheless, to overcome this drawback, we used three and two different prediction models to diagnose NAFLD and advanced fibrosis, respectively, which was likely to improve the accuracy of the results: these models showed fairly reliable performance compared to each other and standard methods [5,12,13]. Further studies are needed to confirm our findings with populations based on the formal criteria for the diagnosis of the diseases.

## 5. Conclusions

In this study, we demonstrated that while circulating retinol and α-tocopherol concentrations had a positive relationship with NAFLD prevalence, advanced fibrosis was only correlated with serum retinol levels. Therefore, in cases of serum retinol deficiency in patients with NAFLD, liver biopsy could be considered to rule out advanced fibrosis. Our findings could provide insight into NAFLD patient care at a micronutrient level.

## Figures and Tables

**Table 1:** Characteristics of subjects according to quartile group for serum retinol.

Total (*n* = 4448)	Serum Retinol				*p*-Value
	Q1 * (*n* = 1112)	Q2 * (*n* = 1112)	Q3 * (*n* = 1112)	Q4 * (*n* = 1112)	
Age (years)	45.9 ± 17.2	49.4 ± 16.1	51.8 ± 15.3	54.2 ± 14.7	<0.001
Sex (male)	210 (18.9)	356 (32.0)	488 (43.9)	623 (56.0)	<0.001
Income					0.04
1st quartile (lowest)	279 (25.1)	295 (26.5)	266 (23.9)	253 (22.8)	
2nd quartile	301 (27.1)	244 (21.9)	269 (24.2)	305 (27.4)	
3rd quartile	266 (23.9)	301 (27.1)	304 (27.3)	270 (24.3)	
4th quartile (highest)	266 (23.9)	272 (24.5)	273 (24.6)	284 (25.5)	
SBP (mmHg)	113.1 ± 16.0	116.8 ± 16.8	119.3 ± 16.6	121.8 ± 16.4	<0.001
BMI (kg/m^2^)	23.1 ± 3.7	23.6 ± 3.7	24.0 ± 3.3	24.4 ± 3.3	<0.001
FBG (mg/dL)	95.4 ± 19.3	98.8 ± 22.1	103.1 ± 28.7	105.0 ± 27.0	<0.001
Total cholesterol (mg/dL)	185.3 ± 32.6	192.0 ± 36.1	195.8 ± 37.4	197.3 ± 40.1	<0.001
Triglycerides (mg/dL)	91.0 ± 56.7	118.9 ± 88.8	134.8 ± 82.9	170.5 ± 119.9	<0.001
HDL-C (mg/dL)	53.4 ± 12.4	51.6 ± 12.9	50.7 ± 12.6	49.5 ± 12.3	<0.001
GFR (mL/min/1.73 m^2^)	95.6 ± 18.2	92.3 ± 17.5	88.5 ± 16.8	84.1 ± 23.5	<0.001
Hypertension	223 (20.1)	291 (26.2)	355 (31.9)	456 (41.0)	<0.001
Diabetes	78 (7.0)	102 (9.2)	154 (13.8)	197 (17.7)	<0.001
Dyslipidemia	282 (25.4)	385 (34.6)	521 (46.9)	634 (57.0)	<0.001
Current smoker	62 (5.6)	107 (9.6)	132 (11.9)	240 (21.6)	<0.001
Alcohol consumption	322 (29.0)	382 (34.4)	431 (38.8)	541 (48.7)	<0.001
Regular exercise	480 (43.2)	485 (43.6)	477 (42.9)	453 (40.7)	0.53
Use of supplements	534 (48.0%)	573 (51.5%)	601 (54.0%)	628 (56.5%)	0.001
Dietary vitamin A intake (μgRE/day)	596.3 ± 677.9	634.6 ± 603.6	620.7 ± 536.4	607.1 ± 493.5	0.81
Serum retinol level (mg/L)	0.32 ± 0.06	0.43 ± 0.03	0.53 ± 0.03	0.74 ± 0.15	<0.001
<0.3 mg/L	325 (29.2)	0 (0)	0 (0)	0 (0)	
0.3–0.7 mg/L	787 (70.8)	1112 (100)	1112 (100)	584 (52.5)	
>0.7 mg/L	0 (0)	0 (0)	0 (0)	528 (47.5)	
NAFLD assessed by HSI	138 (12.4)	193 (17.4)	244 (21.9)	287 (25.8)	<0.001
NAFLD assessed by FSI	151 (13.6)	227 (20.4)	287 (25.8)	429 (38.6)	<0.001
NAFLD assessed by CNS	325 (29.2)	461 (41.5)	578 (52.0)	729 (65.6)	<0.001

Continuous variables are reported as mean with standard deviation, and categorical variables are reported as number with percentage. Serum retinol levels were categorized into quartiles. * Q1, <0.39 mg/L; Q2, 0.39–0.48 mg/L; Q3, 0.48–0.59 mg/L; and Q4, >0.59 mg/L. SBP, systolic blood pressure; BMI, body mass index; HDL-C, high-density lipoprotein cholesterol; GFR, glomerular filtration rate; RE, retinol equivalent; NAFLD, non-alcoholic fatty liver disease; HSI, hepatic steatosis index; FBG, fasting blood glucose; FSI, Framingham steatosis index; CNS, comprehensive NAFLD score. Data are expressed as mean ± standard deviation or number (%).

**Table 2:** Characteristics of subjects according to quartile group for alpha (α)-tocopherol.

Total (*n* = 4448)	α-Tocopherol				*p*-Value
	Q1 * (*n* = 1112)	Q2 * (*n* = 1112)	Q3 * (*n* = 1112)	Q4 * (*n* = 1112)	
Age (years)	46.0 ± 17.7	48.2 ± 16.3	52.5 ± 15.1	54.6 ± 13.7	<0.001
Sex (male)	481 (43.3)	415 (37.3)	403 (36.2)	378 (34.0)	<0.001
Income					0.006
1st quartile (lowest)	311 (28.0)	278 (25.0)	277 (24.9)	227 (20.5)	
2nd quartile	273 (24.6)	279 (25.1)	269 (24.2)	298 (26.8)	
3rd quartile	285 (25.6)	292 (26.2)	271 (24.4)	293 (26.3)	
4th quartile (highest)	243 (21.8)	263 (23.7)	295 (26.5)	294 (26.4)	
SBP (mmHg)	113.9 ± 15.7	116.0 ± 16.4	119.6 ± 17.1	121.5 ± 16.9	<0.001
BMI (kg/m^2^)	23.3 ± 3.7	23.6 ± 3.5	23.9 ± 3.4	24.3 ± 3.4	<0.001
FBG (mg/dL)	98.8 ± 24.0	98.0 ± 19.5	101.5 ± 27.3	104.1 ± 27.4	<0.001
Total cholesterol (mg/dL)	167.4 ± 29.9	186.1 ± 27.9	200.0 ± 32.4	216.8 ± 37.6	<0.001
Triglycerides (mg/dL)	93.7 ± 49.6	110.8 ± 65.3	131.9 ± 71.1	178.7 ± 140.7	<0.001
HDL-C (mg/dL)	49.7 ± 10.8	51.8 ± 12.3	52.1 ± 13.5	51.5 ± 13.6	0.001
GFR (mL/min/1.73 m^2^)	93.6 ± 20.1	91.0 ± 18.4	88.7 ± 18.1	87.0 ± 21.3	<0.001
Hypertension	279 (25.1)	289 (26.0)	369 (33.2)	388 (34.9)	<0.001
Diabetes	139 (12.5)	100 (9.0)	132 (11.9)	160 (14.4)	<0.001
Dyslipidemia	341 (30.7)	346 (31.1)	481 (43.3)	654 (58.8)	<0.001
Current smoker	167 (15.0)	104 (9.4)	131 (11.8)	139 (12.5)	0.003
Alcohol consumption	407 (36.6)	458 (41.2)	419 (37.7)	392 (35.3)	0.03
Regular exercise	471 (42.4)	482 (43.3)	449 (40.4)	493 (44.3)	0.27
Use of supplements	478 (43.0)	530 (47.7)	619 (55.7)	709 (63.8)	<0.001
α-tocopherol level (mg/L)	8.7 ± 1.3	11.4 ± 0.7	13.9 ± 0.9	20.3 ± 5.9	<0.001
<5 mg/L	12 (1.1)	0 (0)	0 (0)	0 (0)	
5–20 mg/L	1100 (98.9)	1112 (100)	1112 (100)	729 (65.6)	
>20 mg/L	0 (0)	0 (0)	0 (0)	383 (34.4)	
NAFLD assessed by HSI	192 (17.3)	184 (16.5)	225 (20.2)	261 (23.5)	<0.001
NAFLD assessed by FSI	206 (18.5)	205 (18.4)	287 (25.8)	396 (35.6)	<0.001
NAFLD assessed by CNS	398 (35.8)	451 (40.6)	572 (51.4)	672 (60.4)	<0.001

Continuous variables are reported as mean with standard deviation, and categorical variables are reported as number with percentage. Serum α-tocopherol levels were categorized into quartiles. * Q1, <10.3 mg/L; Q2, 10.3–12.5 mg/L; Q3, 12.5–15.6 mg/L; and Q4, >15.6 mg/L. SBP, systolic blood pressure; BMI, body mass index; HDL-C, high-density lipoprotein cholesterol; GFR, glomerular filtration rate; NAFLD, non-alcoholic fatty liver disease; HSI, hepatic steatosis index; FBG, fasting blood glucose; FSI, Framingham steatosis index; CNS, comprehensive NAFLD score. Data are expressed as mean ± standard deviation or number (%).

**Table 3:** AORs with 95% CIs for association between serum retinol and NAFLD assessed using different prediction models.

Serum Retinol	NAFLD Assessed by HSI			NAFLD Assessed by FSI			NAFLD Assessed by CNS		
	AOR *	95% CI	*p*-Value	AOR *	95% CI	*p*-Value	AOR *	95% CI	*p*-Value
**All subjects** **(*n* = 4448)**									
Q1	1			1			1		
Q2	1.47	0.99, 2.17	0.06	1.38	0.96, 1.98	0.08	1.69	1.22, 2.34	0.001
Q3	1.79	1.22, 2.61	0.003	1.36	0.96, 1.93	0.08	1.93	1.40, 2.67	<0.001
Q4	1.67	1.22, 2.45	0.01	2.34	1.65, 3.32	<0.001	3.28	2.33, 4.61	<0.001
*p* for trend			0.01			<0.001			<0.001
**Male subjects** **(*n* = 1677)**									
Q1	1			1			1		
Q2	1.13	0.70, 1.82	0.61	0.91	0.58, 1.43	0.70	1.71	1.07, 2.73	0.03
Q3	0.96	0.59, 1.54	0.85	1.00	0.64, 1.55	0.98	2.39	1.49, 3.83	<0.001
Q4	1.10	0.68, 1.78	0.70	2.16	1.39, 3.37	0.001	2.92	1.78, 4.78	<0.001
*p* for trend			0.09			<0.001			<0.001
**Female subjects** **(*n* = 2771)**									
Q1	1			1			1		
Q2	1.16	0.63, 2.15	0.63	1.02	0.60, 1.76	0.94	1.13	0.73, 1.76	0.58
Q3	1.98	1.11, 3.53	0.02	1.48	0.89, 2.46	0.14	1.40	0.91, 2.15	0.13
Q4	2.11	1.17, 3.79	0.01	2.18	1.32, 3.62	0.003	2.59	1.66, 4.05	<0.001
*p* for trend			0.003			<0.001			<0.001

* The logistic model was adjusted for age, sex, BMI, GFR, hypertension, diabetes, dyslipidemia status, income level, smoking status, alcohol consumption, exercise status, use of vitamin supplements, and daily dietary intake of vitamin A. NAFLD, non-alcoholic fatty liver disease; AOR, adjusted odds ratio; CI, confidence interval; HSI, hepatic steatosis index; FSI, Framingham steatosis index; CNS, comprehensive NAFLD score; BMI, body mass index; GFR, glomerular filtration rate.

**Table 4:** AORs with 95% CIs for association between α-tocopherol and NAFLD assessed using different prediction models.

α-Tocopherol	NAFLD Assessed by HSI			NAFLD Assessed by FSI			NAFLD Assessed by CNS		
	AOR *	95% CI	*p*-Value	AOR *	95% CI	*p*-Value	AOR *	95% CI	*p*-Value
**All subjects** **(*n* = 4448)**									
Q1	1			1			1		
Q2	1.14	0.80, 1.63	0.47	1.44	1.02, 2.03	0.04	1.37	0.99, 1.88	0.05
Q3	1.30	0.92, 1.84	0.14	2.08	1.49, 2.91	<0.001	2.32	1.68, 3.20	<0.001
Q4	1.44	1.01, 2.05	0.04	3.96	2.83, 5.56	<0.001	3.39	2.44, 4.71	<0.001
*p* for trend			0.03			<0.001			<0.001
**Male subjects** **(*n* = 1677)**									
Q1	1			1			1		
Q2	1.00	0.62, 1.61	0.99	1.58	1.00, 2.50	0.05	1.02	0.65, 1.62	0.92
Q3	1.41	0.89, 2.23	0.14	1.93	1.24, 3.02	0.004	1.76	1.10, 2.81	0.02
Q4	1.14	0.71, 1.81	0.59	4.34	2.76, 6.84	<0.001	3.87	2.33, 6.84	<0.001
*p* for trend			0.35			<0.001			<0.001
**Female subjects** **(*n* = 2771)**									
Q1	1			1			1		
Q2	1.38	0.76, 2.50	0.28	1.53	0.87, 2.69	0.14	1.71	1.08, 2.70	0.02
Q3	1.72	0.98, 3.04	0.06	2.10	1.24, 3.53	0.006	2.66	1.69, 4.19	<0.001
Q4	2.02	1.14, 3.59	0.02	4.38	2.58, 7.45	<0.001	3.39	2.16, 5.32	<0.001
*p* for trend			0.01			<0.001			<0.001

* The logistic model was adjusted for age, sex, BMI, GFR, hypertension, diabetes, dyslipidemia status, income level, smoking status, alcohol consumption, exercise status, and use of vitamin supplements. NAFLD, non-alcoholic fatty liver disease; AOR, adjusted odds ratio; CI, confidence interval; HSI, hepatic steatosis index; FSI, Framingham steatosis index; CNS, comprehensive NAFLD score; BMI, body mass index; GFR, glomerular filtration rate.

**Table 5:** AORs * with 95% CIs for association of serum retinol and α-tocopherol with advanced fibrosis assessed using BARD and FIB-4.

	NAFLD Assessed by HSI (*n* = 862)						NAFLD Assessed by FSI (*n* = 1094)						NAFLD Assessed by CNS (*n* = 2093)					
	BARD (*n* = 416)			FIB-4 (*n* = 18)			BARD (*n* = 660)			FIB-4 (*n* = 40)			BARD (*n* = 1554)			FIB-4 (*n* = 64)		
	AOR	95% CI	*p*-Value	AOR	95% CI	*p*-Value	AOR	95% CI	*p*-Value	AOR	95% CI	*p*-Value	AOR	95% CI	*p*-Value	AOR	95% CI	*p*-Value
**Serum retinol**																		
Q1	1			1			1			1			1			1		
Q2	0.48	0.29, 0.80	0.01	0.43	0.10, 1.90	0.27	0.58	0.38, 0.89	0.01	0.40	0.15, 1.05	0.06	0.59	0.43, 0.82	0.002	0.68	0.35, 1.34	0.27
Q3	0.39	0.23, 0.65	0.001	0.18	0.03, 1.18	0.07	0.67	0.43, 1.04	0.07	0.28	0.09, 0.83	0.02	0.62	0.44, 0.86	0.004	0.43	0.20, 0.92	0.03
Q4	0.57	0.34, 0.97	0.04	0.12	0.02, 0.83	0.03	1.06	0.67, 1.67	0.82	0.49	0.19, 1.25	0.13	0.80	0.56, 1.13	0.21	0.29	0.12, 0.67	0.004
*p* for trend			0.04			0.02			0.57			0.08			0.39			0.002
**α-tocopherol**																		
Q1	1			1			1			1			1			1		
Q2	0.65	0.40, 1.07	0.09	0.85	0.26, 2.77	0.99	0.89	0.59, 1.36	0.60	0.53	0.21, 1.35	0.18	0.86	0.63, 1.16	0.31	0.51	0.24, 1.08	0.08
Q3	0.98	0.59, 1.63	0.95	1.10	0.27, 4.55	0.89	1.31	0.85, 2.01	0.23	0.65	0.25, 1.66	0.37	0.94	0.69, 1.28	0.68	0.59	0.29, 1.21	0.15
Q4	0.88	0.53, 1.46	0.61	0.46	0.10, 2.25	0.34	1.51	0.97, 2.35	0.07	0.62	0.23, 1.66	0.34	0.95	0.69, 1.31	0.77	0.56	0.26, 1.19	0.13
*p* for trend			0.94			0.75			0.02			0.36			0.91			0.14

* The logistic model was adjusted for age, sex, BMI, GFR, hypertension, diabetes, dyslipidemia status, income level, smoking status, alcohol consumption, exercise status, use of vitamin supplements, and daily dietary intake of vitamin A. NAFLD, non-alcoholic fatty liver disease; AOR, adjusted odds ratio; CI, confidence interval; HSI, hepatic steatosis index; FSI, Framingham steatosis index; CNS, comprehensive NAFLD score; BMI, body mass index; GFR, glomerular filtration rate.

## Data Availability

Data are available from the seventh Korea National Health and Nutrition Examination Survey (KNHANES VII, 2016–2018), conducted by the Korea Centers for Disease Control and Prevention (KCDCP). Data are freely available from KCDCP after signing up (https://knhanes.cdc.go.kr, accessed on 14 December 2020).

## Supplementary Materials

The following are available online at https://www.mdpi.com/article/10.3390/nu13051720/s1, Figure S1: Proportion of subjects with serum retinol and α-tocopherol levels according to quartile with predicted NAFLD prevalence (FSI) by quartile, Figure S2: Proportion of subjects with serum retinol and α-tocopherol levels according to quartile with predicted NAFLD prevalence (CNS) by quartile, Figure S3: Proportion of subjects with serum retinol levels according to reference value with NAFLD scores, Figure S4: Proportion of subjects with serum retinol levels according to reference values for advanced fibrosis (assessed by BARD and FIB-4), Table S1: Prediction models for NAFLD and advanced fibrosis, Table S2: AORs with 95% CIs for association of serum retinol with NAFLD assessed using different prediction models (all subjects including those with HBV, HCV, and liver cirrhosis and heavy alcoholics for the sensitivity analysis), Table S3: AORs with 95% CIs for association of α-tocopherol with NAFLD assessed using different prediction models (all subjects including those with HBV, HCV, and liver cirrhosis and heavy alcoholics for the sensitivity analysis), Table S4: AORs with 95% CIs for association between α-tocopherol with cholesterol adjustment and NAFLD assessed using different prediction models, Table S5: AORs with 95% CIs for association between α-tocopherol with cholesterol adjustment and advanced fibrosis assessed using BARD and FIB-4.
