# Supplementary material for: Dynamics of Serum Retinol and Alpha-Tocopherol Levels According to Non-Alcoholic Fatty Liver Disease Status
Source: Nutrients. 2021 May 19;13(5):1720. doi: 10.3390/nu13051720 (PMC8161312; doi:10.3390/nu13051720)
Supplement: Supplementary file 1 [file nutrients-13-01720-s001.zip › nutrients-1197567-supplementary.pdf]

Supplementary Table S1. Prediction models for NAFLD and advanced fibrosis

NAFLD prediction models	Cutoff values	Equation
Hepatic steatosis index (HSI)	> 36	HSI = 8×ALT/AST ratio + BMI (+2, if diabetic; +2, if female)
Framingham steatosis index (FSI)	≥ 23	FSI = $\frac{1}{1+e^{-x}} \times 100$<br>where x = -7.981 + 0.011 × age (years) – 0.146 × sex (female=1, male=0) + 0.173 × BMI (kg/m^2) + 0.007 × triglycerides (mg/dL) + 0.593 × hypertension (yes=1, no=0) + 0.789 × diabetes (yes=1, no=0) + 1.1 × ALT:AST ratio ≥ 1.33 (yes=1, no=0)
Comprehensive NAFLD score (CNS)	≥ 40	CNS = $\frac{1}{1+e^{-x}} \times 100$<br>If male, x = 0.016 × age (years) + 0.182 × BMI (kg/m^2) + 0.089 × waist circumference (cm) + 0.391 × alcohol (yes=1, no=0) + 0.124 × exercise (yes=0, no=1) + 0.018 × fasting glucose (mg/dL) + 0.773 × $\log_e$(triglycerides [mg/dL]) – 0.014 × HDL cholesterol (mg/dL) + 0.145 × uric acid (mg/dL) – 0.674 × $\log_e$(AST [IU/L]) + 1.632 × $\log_e$(ALT [IU/L]) – 21.695.<br><br>If female, x = 0.320 × BMI (kg/m^2) + 0.044 × waist circumference (cm) + 0.533 × diabetes (yes=1, no=0) + 0.016 × fasting glucose (mg/dL) + 0.951 × $\log_e$(triglycerides [mg/dL]) – 0.015 × HDL cholesterol (mg/dL) + 0.199 × uric acid (mg/dL) – 0.645 × $\log_e$(AST [IU/L]) + 1.302 × $\log_e$(ALT [IU/L]) + 0.255 × menopause (yes=1, no=0) – 19.741.
Advanced fibrosis prediction models		
BARD score	≥ 2	BARD = weighted sum (BMI > 28 = 1 point, AST/ALT ratio > 0.8 = 2 points, diabetes = 1 point)
FIB-4 index	≥ 2.67	FIB-4 = age (years) $\times \frac{\text{AST (IU/L)}}{\text{platelets } (10^9/\text{L}) \times \sqrt{\text{ALT (IU/L)}}}$

NAFLD, non-alcoholic fatty liver disease; BMI, body mass index; IU, international units; ALT, alanine aminotransferase; AST, aspartate aminotransferase.

Supplementary Table S2. AORs with 95% CIs for association of serum retinol with NAFLD assessed using different prediction models (all subjects including those with HBV, HCV, and liver cirrhosis and heavy alcoholics for the sensitivity analysis).

	NAFLD assessed by HSI			NAFLD assessed by FSI			NAFLD assessed by CNS		
Serum retinol	AOR *	95% CI	p-value	AOR *	95% CI	p-value	AOR *	95% CI	p-value
All subjects (n = 5,373)									
Q1	1			1			1		
Q2	1.28	0.90, 1.81	0.17	1.38	1.00, 1.90	0.05	1.52	1.14, 2.02	0.004
Q3	1.51	1.08, 2.11	0.02	1.49	1.10, 2.03	0.01	2.03	1.53, 2.70	<0.001
Q4	1.61	1.15, 2.27	0.006	2.65	1.94, 3.62	<0.001	3.09	2.29, 4.18	<0.001
P for trend			0.005			<0.001			<0.001
Male subjects (n =2,337)									
Q1	1			1			1		
Q2	1.01	0.60, 1.72	0.96	1.13	0.70, 1.83	0.63	2.58	1.59, 4.20	<0.001
Q3	1.02	0.62, 1.68	0.93	1.21	0.76, 1.91	0.42	3.40	2.13, 5.42	<0.001
Q4	1.18	0.72, 1.91	0.51	2.13	1.36, 3.32	0.001	4.59	2.90, 7.27	<0.001
P for trend			0.40			<0.001			<0.001
Female subjects (n =3,036)									
Q1	1			1			1		
Q2	1.43	0.86, 2.38	0.17	1.63	1.04, 2.55	0.03	1.20	0.83, 1.73	0.33
Q3	1.86	1.13, 3.05	0.01	1.59	1.03, 2.46	0.04	1.55	1.05, 2.28	0.03
Q4	1.89	1.10, 3.25	0.02	2.91	1.82, 4.66	<0.001	2.70	1.73, 4.22	<0.001
P for trend			0.01			<0.001			<0.001

*The logistic model was adjusted for age, sex, BMI, GFR, hypertension, diabetes mellitus, dyslipidemia status, income level, smoking status, alcohol consumption, exercise status, use of vitamin supplements, and daily dietary intake of vitamin A. NAFLD, non-alcoholic fatty liver disease; AOR, adjusted odds ratio; CI, confidence interval; HSI, hepatic steatosis index; FSI, Framingham steatosis index; CNS, comprehensive NAFLD score; BMI, body mass index; GFR, glomerular filtration rate.

Supplementary Table S3. AORs with 95% CIs for association of α-tocopherol with NAFLD assessed using different prediction models (all subjects including those with HBV, HCV, and liver cirrhosis and heavy alcoholics for the sensitivity analysis).

	NAFLD assessed by HSI			NAFLD assessed by FSI			NAFLD assessed by CNS		
α-tocopherol	AOR*	95% CI	P-value	AOR*	95% CI	P-value	AOR*	95% CI	P-value
All subjects (n = 5,373)									
Q1	1			1			1		
Q2	1.19	0.86, 1.64	0.29	1.52	1.11, 2.07	0.008	1.32	1.00, 1.76	0.05
Q3	1.37	1.00, 1.87	0.05	2.10	1.55, 2.83	<0.001	2.07	1.56, 2.74	<0.001
Q4	1.42	1.03, 1.94	0.03	4.40	3.25, 5.95	<0.001	3.14	2.35, 4.21	<0.001
P for trend			0.02			<0.001			<0.001
Male subjects (n =2,337)									
Q1	1			1			1		
Q2	1.22	0.82, 1.83	0.33	1.42	0.97, 2.07	0.07	1.11	0.77, 1.61	0.58
Q3	1.20	0.81, 1.78	0.37	1.99	1.37, 2.88	<0.001	1.79	1.22, 2.61	0.003
Q4	1.15	0.76, 1.73	0.51	4.12	2.82, 6.03	<0.001	3.51	2.30, 5.37	<0.001
P for trend			0.56			<0.001			<0.001
Female subjects (n =3,036)									
Q1	1			1			1		
Q2	1.34	0.76, 2.38	0.32	1.76	1.00, 3.08	0.05	1.64	1.05, 2.56	0.03
Q3	1.95	1.12, 3.39	0.02	2.37	1.40, 4.02	0.001	2.38	1.53, 3.70	<0.001
Q4	2.28	1.31, 3.96	0.003	5.43	3.19, 9.24	<0.001	3.27	2.12, 5.06	<0.001
P for trend			0.001			<0.001			<0.001

*The logistic model was adjusted for age, sex, BMI, GFR, hypertension, diabetes mellitus, dyslipidemia status, income level, smoking status, alcohol consumption, exercise status, and use of vitamin supplements. NAFLD, non-alcoholic fatty liver disease; AOR, adjusted odds ratio; CI, confidence interval; HSI, hepatic steatosis index; FSI, Framingham steatosis index; CNS, comprehensive NAFLD score; BMI, body mass index; GFR, glomerular filtration rate.

Supplementary Table S4. AORs with 95% CIs for association between α-tocopherol with cholesterol adjustment and NAFLD assessed using different prediction models.

α-tocopherol/cholesterol ratio	NAFLD assessed by HSI			NAFLD assessed by FSI			NAFLD assessed by CNS		
	AOR*	95% CI	P-value	AOR*	95% CI	P-value	AOR*	95% CI	P-value
All subjects (n = 4,448)									
Q1	1			1			1		
Q2	0.87	0.61, 1.24	0.44	1.27	0.91, 1.78	0.16	1.41	1.04, 1.92	0.03
Q3	1.16	0.81, 1.64	0.42	1.51	1.09, 2.11	0.01	1.64	1.20, 2.23	0.002
Q4	1.03	0.72, 1.47	0.88	2.37	1.71, 3.30	<0.001	2.19	1.59, 3.01	<0.001
P for trend			0.53			<0.001			<0.001
Male subjects (n = 1,677)									
Q1	1			1			1		
Q2	0.92	0.59, 1.45	0.73	1.43	0.93, 2.20	0.11	1.29	0.83, 2.00	0.25
Q3	0.90	0.57, 1.44	0.67	1.71	1.11, 2.66	0.02	1.59	1.00, 2.54	0.05
Q4	0.75	0.47, 1.20	0.23	2.26	1.45, 3.54	<0.001	1.71	1.05, 2.77	0.03
P for trend			0.25			<0.001			0.02
Female subjects (n =2,771)									
Q1	1			1			1		
Q2	0.87	0.47, 1.59	0.87	1.02	0.59, 1.76	0.94	1.59	1.02, 2.46	0.04
Q3	2.13	1.19, 3.84	0.01	1.32	0.78, 2.23	0.30	1.75	1.13, 2.69	0.01
Q4	1.79	1.00, 3.22	0.05	2.32	1.39, 3.88	0.001	2.59	1.66, 4.02	<0.001
P for trend			0.006			<0.001			<0.001

α-tocopherol with the cholesterol adjustment was defined as vitamin E/cholesterol ratio. *The logistic model was adjusted for age, sex, BMI, GFR, hypertension, diabetes mellitus, dyslipidemia status, income level, smoking status, alcohol consumption, exercise status, and use of vitamin supplements. NAFLD, non-alcoholic fatty liver disease; AOR, adjusted odds ratio; CI, confidence interval; HSI, hepatic steatosis index; FSI, Framingham steatosis index; CNS, comprehensive NAFLD score; BMI, body mass index; GFR, glomerular filtration rate.

Supplementary Table S5. AORs with 95% CIs for association between α-tocopherol with cholesterol adjustment and advanced fibrosis assessed using BARD and FIB-4.

	NAFLD assessed by HSI (n = 862)						NAFLD assessed by FSI (n = 1,094)						NAFLD assessed by CNS (n = 2,093)					
	BARD (n = 416)			FIB-4 (n = 18)			BARD (n = 660)			FIB-4 (n = 40)			BARD (n = 1,554)			FIB-4 (n = 64)		
α-tocopherol/cholesterol ratio	AOR	95% CI	P-value	AOR	95% CI	P-value	AOR	95% CI	P-value	AOR	95% CI	P-value	AOR	95% CI	P-value	AOR	95% CI	P-value
Q1	1			1			1			1			1			1		
Q2	0.85	0.50, 1.43	0.54	0.45	0.10, 2.07	0.30	1.17	0.77, 1.76	0.46	0.93	0.32, 2.72	0.89	1.09	0.81, 1.47	0.57	1.41	0.60, 3.27	0.43
Q3	0.73	0.45, 1.18	0.20	0.26	0.04, 1.52	0.14	1.32	0.87, 2.00	0.19	1.05	0.37, 2.99	0.93	0.98	0.72, 1.33	0.91	0.97	0.41, 2.28	0.94
Q4	0.93	0.58, 1.50	0.77	0.23	0.04, 1.25	0.09	0.77	0.50, 1.18	0.23	2.14	0.81, 5.68	0.13	1.13	0.82, 1.56	0.46	1.90	0.86, 4.20	0.11
P for trend			0.35			0.24			0.32			0.10			0.62			0.17

α-tocopherol with the cholesterol adjustment was defined as vitamin E/cholesterol ratio. *The logistic model was adjusted for age, sex, BMI, GFR, hypertension, diabetes, dyslipidemia status, income level, smoking status, alcohol consumption, exercise status, and use of vitamin supplements. NAFLD, non-alcoholic fatty liver disease; AOR, adjusted odds ratio; CI, confidence interval; HSI, hepatic steatosis index; FSI, Framingham steatosis index; CNS, comprehensive NAFLD score; BMI, body mass index; GFR, glomerular filtration rate.

Supplementary Figure S1. Proportion of subjects with serum retinol and α-tocopherol levels according to quartile with predicted NAFLD prevalence (FSI) by quartile.

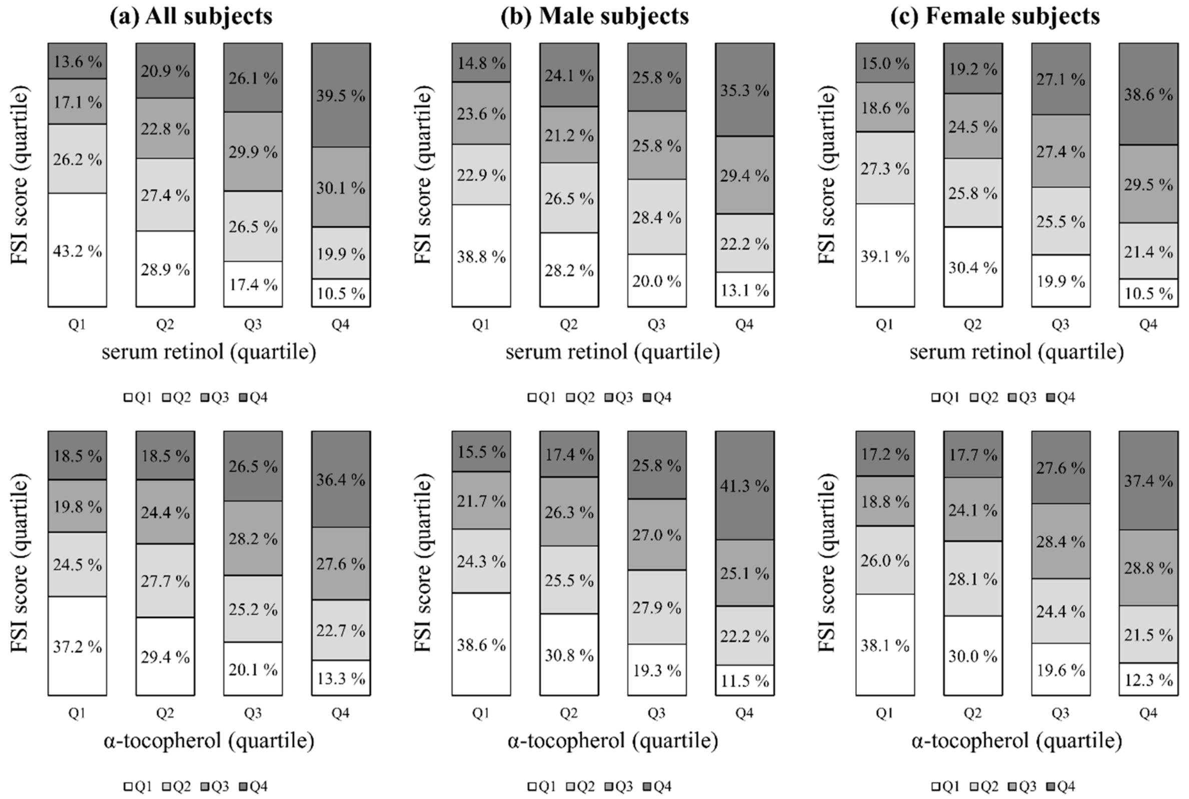

The study population was divided into quartile groups according to NAFLD (FSI) and vitamin (serum retinol and α-tocopherol) levels, respectively. Serum retinol and α-tocopherol had strong positive associations with the NAFLD (FSI) prediction model (Ps < 0.0001 for the trends). NAFLD, non-alcoholic fatty liver disease; FSI, Framingham steatosis index; Q, quartile.

Supplementary Figure S2. Proportion of subjects with serum retinol and α-tocopherol levels according to quartile with predicted NAFLD prevalence (CNS) by quartile.

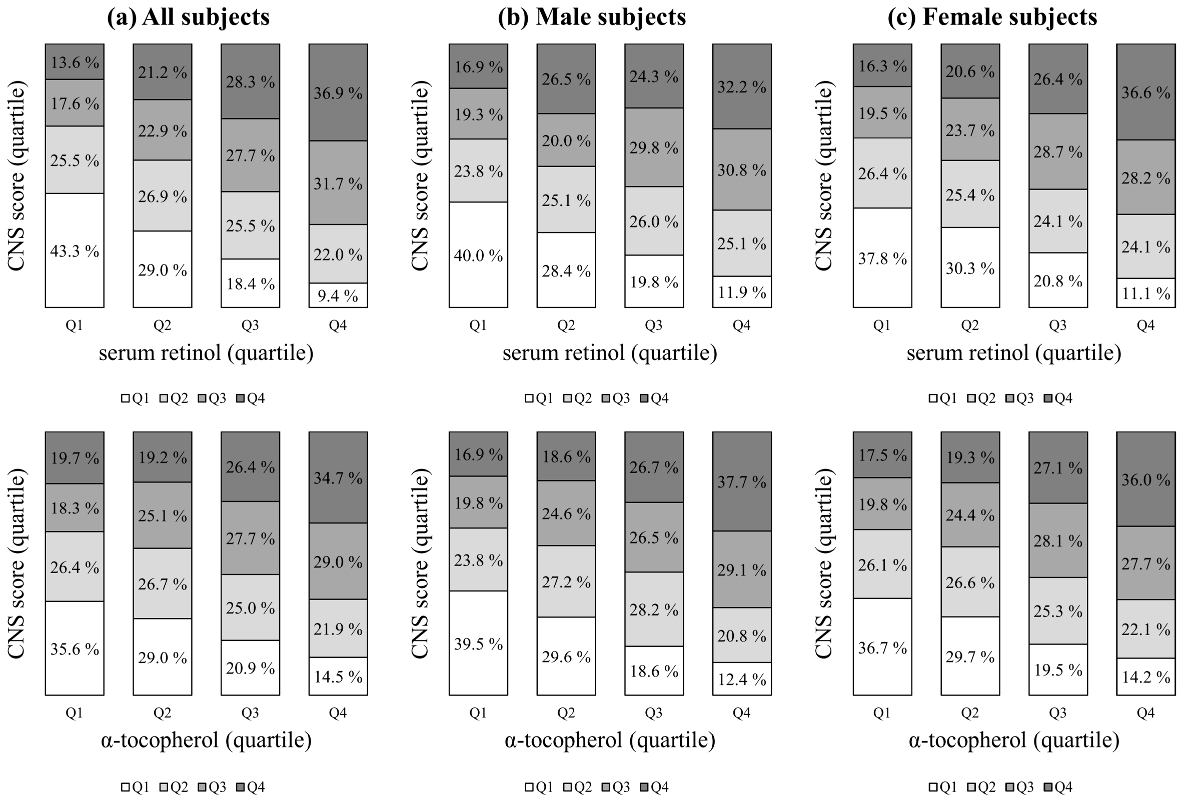

The study population was divided into quartile groups according to NAFLD (CNS) and vitamin (serum retinol and α-tocopherol) levels, respectively. Serum retinol and α-tocopherol had strong positive associations with the NAFLD (CNS) prediction model (Ps < 0.0001 for the trends). NAFLD, non-alcoholic fatty liver disease; CNS, comprehensive NAFLD score; Q, quartile.

Supplementary Figure S3. Proportion of subjects with serum retinol levels according to reference value with NAFLD scores.

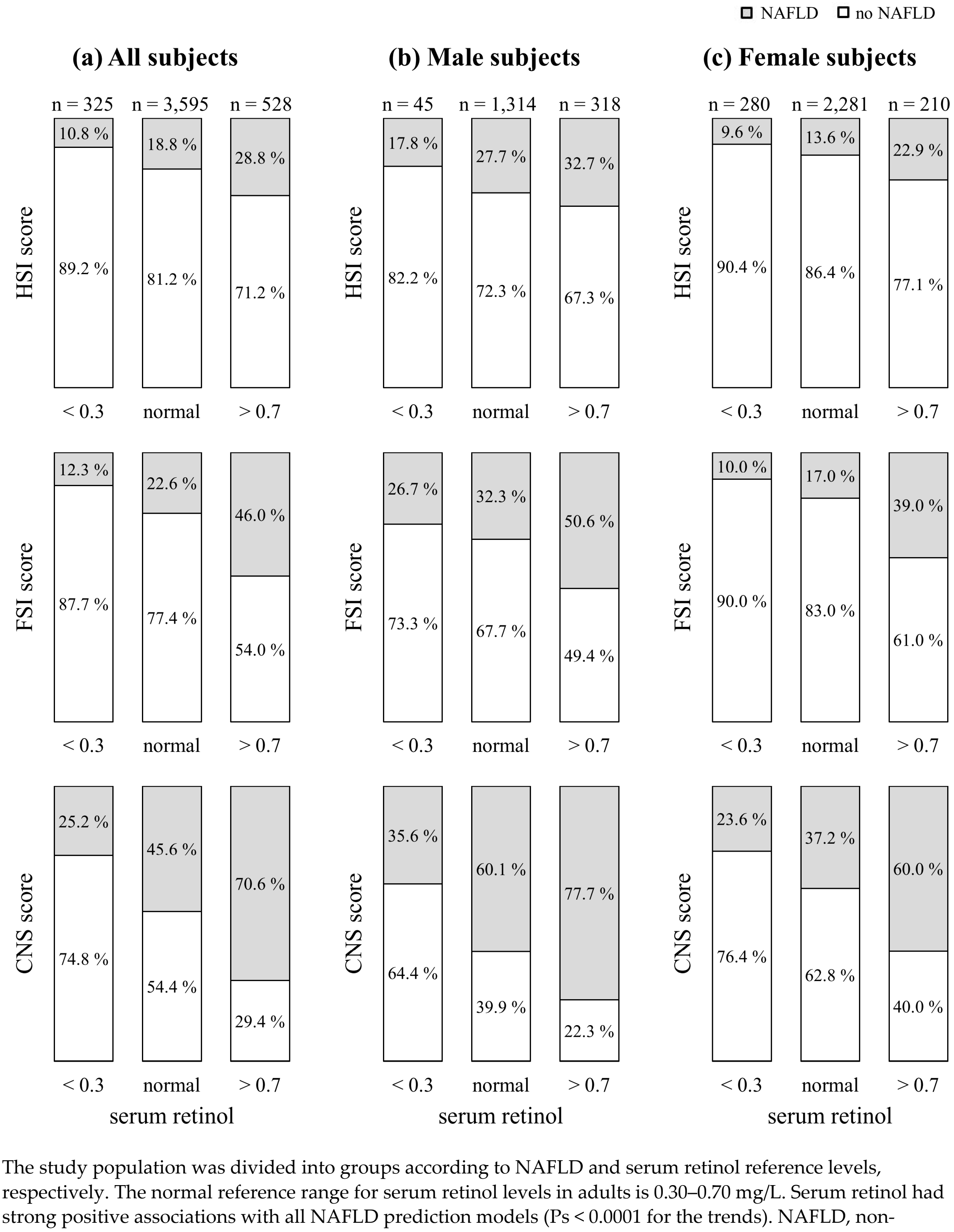

The study population was divided into groups according to NAFLD and serum retinol reference levels, respectively. The normal reference range for serum retinol levels in adults is 0.30–0.70 mg/L. Serum retinol had strong positive associations with all NAFLD prediction models (Ps < 0.0001 for the trends). NAFLD, non-alcoholic fatty liver disease; HSI, hepatic steatosis index; FSI, Framingham steatosis index; CNS, comprehensive NAFLD score.

Supplementary Figure S4. Proportion of subjects with serum retinol levels according to reference values for advanced fibrosis (assessed by BARD and FIB-4).

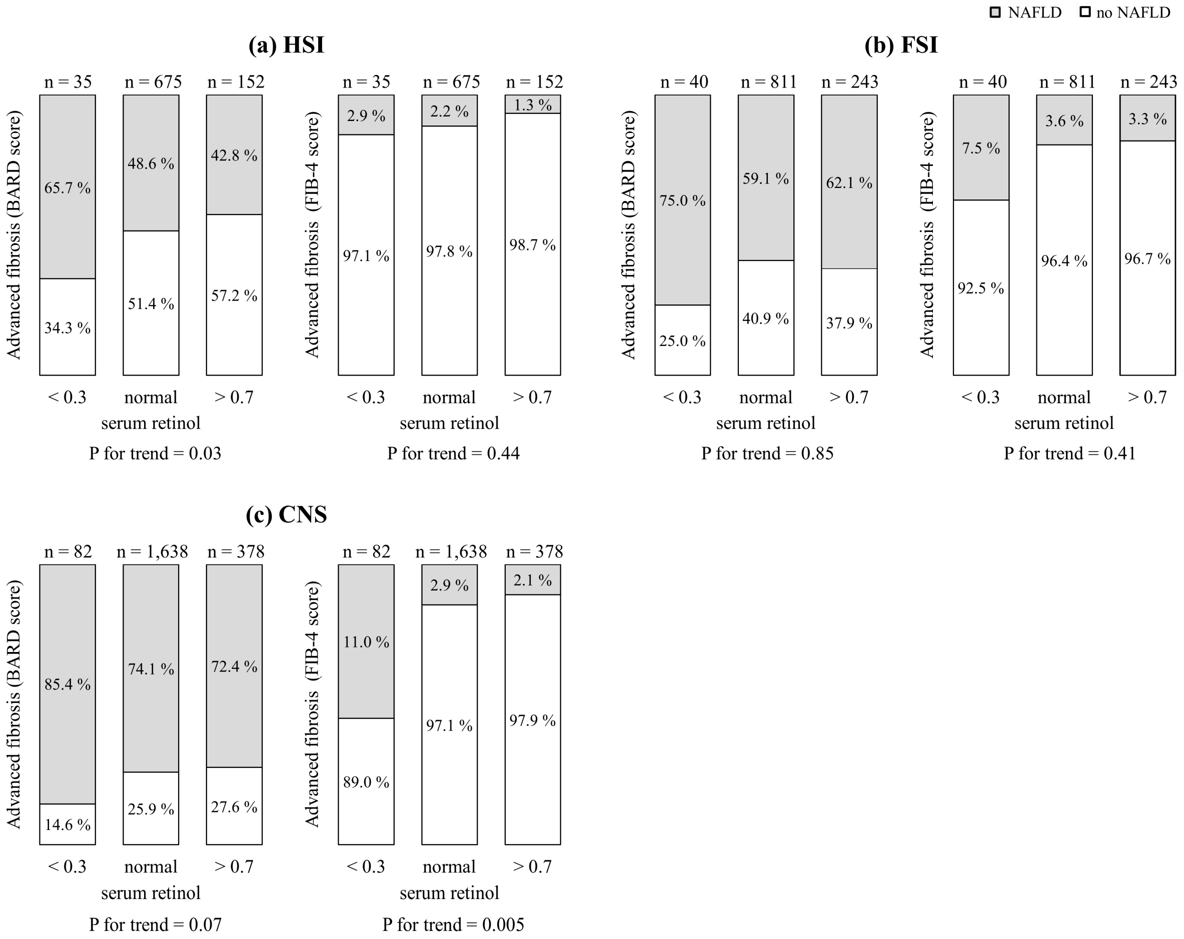

The study population for NAFLD was divided into groups according to advanced fibrosis and serum retinol reference levels, respectively. The normal reference range for serum retinol levels in adults is 0.30–0.70 mg/L. NAFLD, non-alcoholic fatty liver disease; HSI, hepatic steatosis index; FSI, Framingham steatosis index; CNS, comprehensive NAFLD score.
